# The typical polypoid adenomyoma is a special form of endometrial polyp: a case-controlled study with a large sample size

**DOI:** 10.1186/s40001-023-01286-1

**Published:** 2023-08-30

**Authors:** Xinmei Wang, Hongyuan Zhang, Juan Xu, Pengpeng Qu

**Affiliations:** Department of Gynecological Oncology, Tianjin Central Hospital of Gynecology and Obstetrics, No. 156, Nankaisan Road, Tianjin, 300100 China

**Keywords:** Endometrial injury, Endometrial polyps, Estrogen, Hysteroscopic electrotomy, Typical polypoid adenomyoma

## Abstract

**Purpose:**

To investigate clinicopathological differences between typical endometrial polypoid adenomyomas (TPAs) and endometrial polyps (EPs) and to determine the risk factors and recurrence of TPA and further clarify the pathogenesis and treatment of TPA.

**Methods:**

We reviewed the medical records of 488 women with TPA and 500 women with EP. Then, we analyzed the clinical features and manifestations, ultrasonic manifestations, hysteroscopic morphology, and pathological results. In addition, 360 cases of TPA and 367 cases of EP were followed up for 22–77 months and the risk factors TPA recurrence were assessed.

**Results:**

We detected significant differences in age, menopausal status, body mass index (BMI), the number of pregnancies, and parity between the two groups (*P* < 0.05). Hysteroscopy revealed that the incidence of polyps > 3 cm in diameter and multiple polyps in the TPA group was significantly higher than that in the EP group (*P* < 0.01). In addition, the rate of recurrence in the TPA group was significantly higher than that in the EP group (*P* < 0.05). Over three pregnancies, menopause, curettage, and the application of polyp clamps were all identified as independent risk factors for the recurrence of TPA (*P* < 0.05).

**Conclusion:**

In addition to high estrogen levels, endometrial injury was identified as the main contributor to TPA pathogenesis. Hysteroscopic electrotomy was identified as the preferential treatment for TPA to avoid recurrence, especially in women with risk factors. Increasing the depth of ablation may prevent the recurrence of TPA more efficiently.

## Introduction

Endometrial typical polypoid adenomyomas (TPAs), also known as adenomyomatous polyps, are rarely detected and represent a benign form of neoplasm in the fibroid stroma and glands for which the histological origin remains unclear [1]. TPA accounts for 1.3% of all endometrial polyps (EP) [2]. Histologically, endometrial adenomyomas can be divided into TPAs and atypical polypoid adenomyomas (APA) [3, 4]. TPAs mainly involve normal endometrial glands with interwoven smooth muscle stroma [5, 6]. In contrast, the APAs are lesions with unspecified malignant potential due to a crowded, irregular, and complex glandular epithelium along with abundant, chaotic, and lose smooth muscle stroma, which can resemble atypical endometrial hyperplasia [7, 8].

With the development of hysteroscopy, the detection rate of EP has increased significantly and researchers have come to realize that TPA differs from common endometrial polyps [9]. The lack of systematic analysis of relevant clinical data relating to the pathogenesis of TPA means that we have an insufficient understanding of TPA. In this study, the clinical data of 488 cases of TPA and 500 cases of EP were analyzed retrospectively. We also followed up 360 cases of TPA and 367 cases of EP for 22–77 months. Finally, the specific clinicopathological features and recurrence of endometrial TPA were investigated, and the pathogenesis and treatment were further defined.

## Clinical data and methods

### Clinical data

This study included 988 women who were admitted to the Outpatient or Inpatient Departments of Tianjin Central Obstetrics and Gynecology Hospital between January 2011 and December 2017. The inclusion criteria were as follows: (1) patients who had not received hormone therapy within the previous six months; (2) patients who had a definite pathological diagnosis, and (3) patients with a complete set of data. The exclusion criteria were as follows: (1) patients with malignant tumors; (2) patients with mental or psychological diseases, and (3) patients with an incomplete set of data.

We recorded a range of data for each patient, including age, body mass index (BMI), reproductive history, and surgical method. Two experienced hospital pathologists re-reviewed the pathological specimens and made a suitable diagnosis. According to the pathological results, the patients were divided into two groups: a TPA group (*n* = 488) and an EP group (*n* = 500). Then, we analyzed the clinical features and manifestations, ultrasonic manifestations, hysteroscopic morphology, and pathological results. In addition, 360 cases of TPA and 367 cases of EP were followed up for 22–77 months. The long-term postoperative recurrence rate of patients in the two groups was then compared and the risk factors for the recurrence of TPA were assessed.

Patient anonymity was preserved as the data were collected from the hospital's electronic medical records. In addition, the research ethics committee of Tianjin Central Hospital of Gynecology and Obstetrics waived the requirement for ethics approval and informed consent because the study used previously stored data.

### Ultrasonography

The ultrasonography was performed by a Japanese Aloka SSD-1100 fan-scan ultrasound instrument with a probe frequency of 3.5 MHz and was performed vaginally.

### Hysteroscope

Hysteroscope was performed by Olympus OES4000 endoscopic system and Johnson & Johnson rigid hysteroscope.

### Hysteroscopic surgery

The perfusion fluid was treated with 0.9% normal saline, and the swelling pressure was 60–80 mmHg. The cutting power was 80W. All the polypoid tissue was removed by ring electrode to the endometrium layer, and the postoperative specimens were sent for pathological examination.

### Statistical methods

SPSS version 28.0 software was used for all statistical analyses (SPSS Inc, Chicago, IL, USA). The Chi-squared test was used for comparisons. Multivariate analysis was performed using a logistic regression model. The log-rank test was used to study the long-term postoperative recurrence rate. The Kaplan–Meier method was used to plot a cumulative recurrence curve. All tests were two-sided, with the significance threshold at *P* < 0.05.

## Results

### Relationship between clinical characteristics and TPA

The mean age of patients in the TPA group was 53.54 ± 3.19 years while the mean age of patients in the EP group was 49.34 ± 4.23 years. The mean BMI of patients in the TPA group was 25.31 ± 4.17 kg/m^2^ while the mean BMI of patients in the EP group was 22.38 ± 3.21 kg/m^2^. The mean gravidity and parity of patients in the TPA group were 3.22 and 1.94, respectively; this compared to 2.22 and 1.14, respectively, in the EP group. We detected significant differences in age, menopausal status, BMI, gravidity, and parity between the two groups (*P* < 0.05). Furthermore, the proportions of patients with an age > 50 years, menopause, obesity, a gravidity > 3, and a parity > 2 in the TPA group were significantly higher than those in the EP group (*P* < 0.05; Table 1).

**Table 1:** Univariate and multivariate analyses of the clinical characteristics of TPA

Parameter	TPA group (*n*, %)	EP group (*n*, %)	Univariate analysis	Multivariate analysis	
			*P*-value	OR (95% CI)	*P*-value
Age (years)					
≤ 50	189 (38.73)	266 (53.2)	0.021	1.41 (1.343–3.224)	0.031
> 50	299 (61.27)	234 (46.8)			
Menopause					
No	156 (31.97)	291 (58.2)	< 0.001	0.337 (0.260–0.438)	< 0.001
Yes	332 (68.03)	209 (41.8)			
BMI					
Normal	146 (29.92)	186 (37.2)	0.011	0.801 (0.370–0.955)	0.032
Overweight	255 (52.25)	223 (44.6)			
Obese	87 (17.83)	91 (18.2)			
Gravidity					
≤ 3	197 (40.39)	302 (60.4)	< 0.001	0.444 (0.344–0.573)	0.001
> 3	291 (59.63)	198 (39.6)			
Parity					
≤ 2	201 (41.18)	240 (48)	0.031	0.759 (0.590–0.976)	0.037
> 2	287 (58.81)	60 (52)			

TPA, typical endometrial polypoid adenomyoma; BMI, body mass index; OR, odds ratio; CI, confidence interval

### Clinical manifestations

In the TPA group, 288 cases (59.01%) had abnormal bleeding and 142 cases had menstrual changes. Other manifestations were secondary dysmenorrhea in 23 cases, vaginal discharge in three cases and infertility in four cases. Another 28 cases were asymptomatic. Compared to patients in the EP group, patients with TPA were more likely to present with clinical manifestations such as irregular vaginal bleeding, menorrhagia, menostaxis, and dysmenorrhea (*P* < 0.05) (Table 2).

**Table 2:** Clinical manifestations in TPA group and EP group

Clinical manifestations		TPA group (*n*, %)	EP group (*n*, %)	Chi-squared	*P*-value
Abnormal bleeding	Irregular vaginal bleeding	268 (54.9)	242 (45.1)	4.201	0.042
	Vaginal bleeding after sexual intercourse	20 (40.00)	30 (60.00)	1.859	0.111
Menstrual changes	Menorrhagia	68 (66.67)	34 (33.33)	13.578	0.000
	Menostaxis	33 (52.38)	30 (47.62)	10.168	0.001
	Shorter menstrual cycle	23 (51.11)	22 (48.89)	0.056	0.467
	Menstrual disturbances	18 (48.65)	19 (51.35)	0.009	0.530
Other symptoms	Dysmenorrhea	23 (85.19)	4 (14.81)	14.227	0.000
	Vaginal discharge	3 (60.00)	2 (40.00)	0.226	0.489
	Infertility	4 (57.14)	3 (42.86)	0.169	0.487
Asymptomatic		28 (19.72)	114 (80.28)	58.420	0.000
Total		488 (49.39)	500 (50.61)		

### Transvaginal ultrasound

**Fig. 1:**
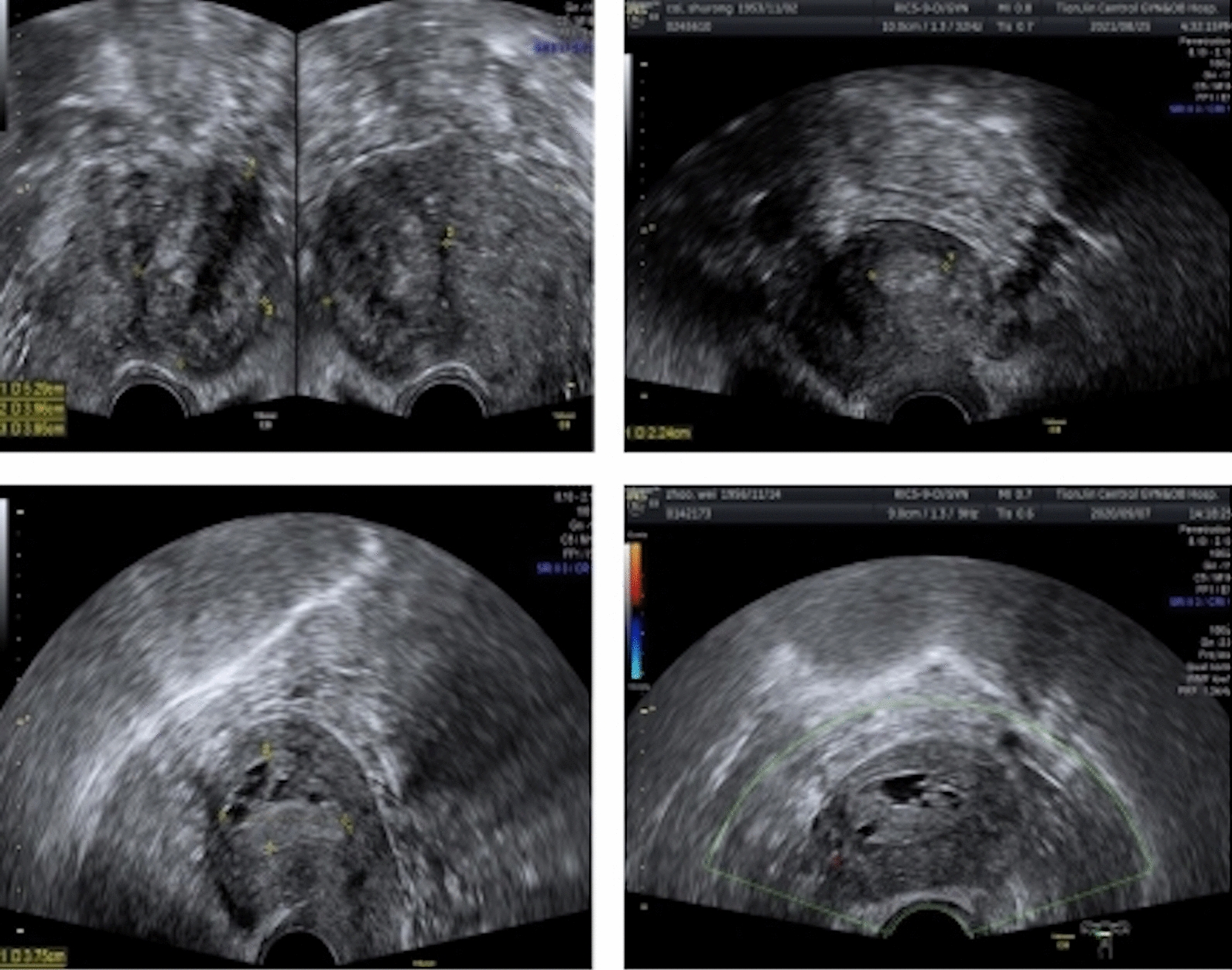
Transvaginal ultrasound image of TPA

All patients received preoperative transvaginal ultrasound examination and 384 cases showed occupying lesions with clear boundaries in the uterine cavity or cervical canal. Twenty-nine cases had pedicled polyps in the lower segment of the uterine cavity which protruded out of the cervical orifice. Of the 384 patients with occupying lesions, 280 (72.92%; 228/384) showed strong or partial echoes in the uterine cavity or cervical canal while 104 patients (27.08%; 104/384) showed low-intensity echoes in the uterine cavity or cervical canal. There were 233 cases (60.67%, 233/384) with striated blood flow signals that were suggestive of a pedicle structure. The occupying space contained multiple small anechoic regions with uneven echoes in 229 cases (59.55%; 229/384) while dark liquid areas were detected in 44 patients with occupying lesions (11.45%; 44/384) (Fig. 1).

### Hysteroscopy

**Fig. 2:**
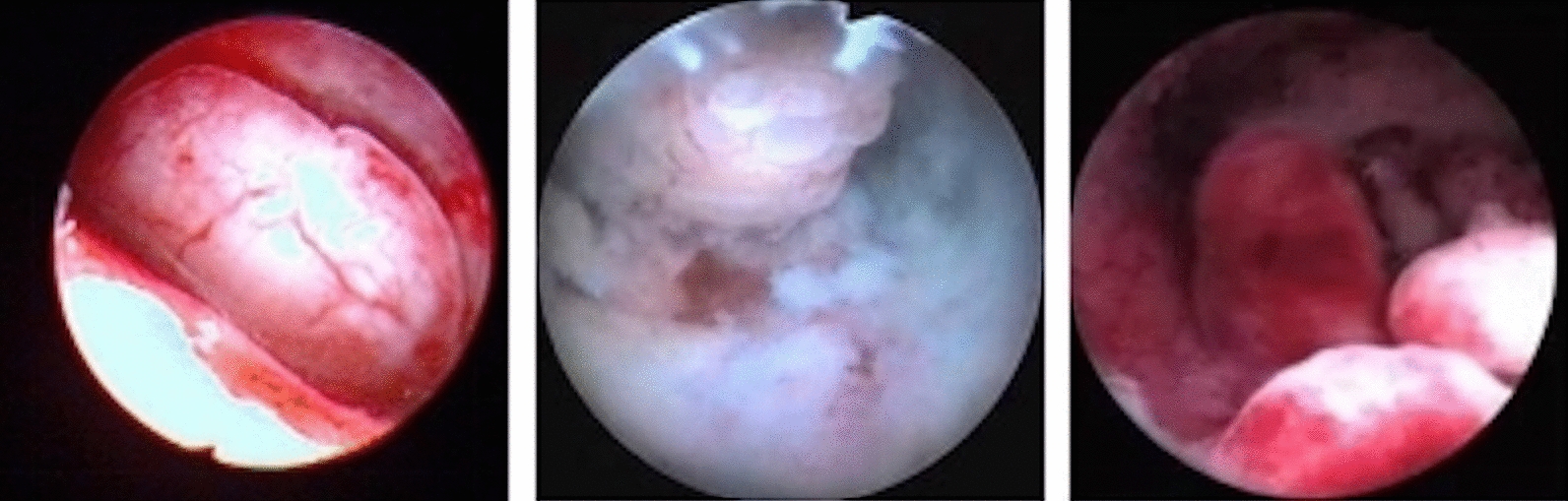
Presentation of TPA under hysteroscopy

Hysteroscopy was performed on 360 patients in the TPA group and 367 patients in the EP group. Under hysteroscopy, it was found that TPA masses were polypoid or nodular with a pink or pale surface; lesion diameter ranged from 0.3 to 6 cm (Fig. 2). The proportion of patients with a lesion diameter > 3 cm and multiple polyps in the TPA group was significantly higher than in the EP group. Compared with patients with EPs, TPA was more likely to exist in the posterior wall (52.95%;193/367), followed by the anterior wall and the lower segment of the uterine cavity (*P* < 0.05) (Table 3).

**Table 3:** The characteristics of TPA and EP under hysteroscopy

Hysteroscopy characters	TPA group (*n*, %)	EP group (*n*, %)	Chi-squared	*P*-value
Size (cm)				
≤ 3	203 (56.4)	243 (66.2)	7.396	0.008
> 3	157 (43.6)	124 (33.8)		
Number				
Single	322 (89.4)	346 (94.3)	5.694	0.012
Multiple	38 (10.6)	21 (5.7)		
Position				
Posterior wall	193 (56.3)	137 (37.3)	41.517	0.000
Anterior wall	91 (25.3)	81 (22.1)		
Lower segment	33 (9.2)	34 (9.3)		
Left/right side wall	19 (5.3)	28 (7.6)		
Others	24 (6.7)	87 (23.7)		

### Pathology

**Fig. 3:**
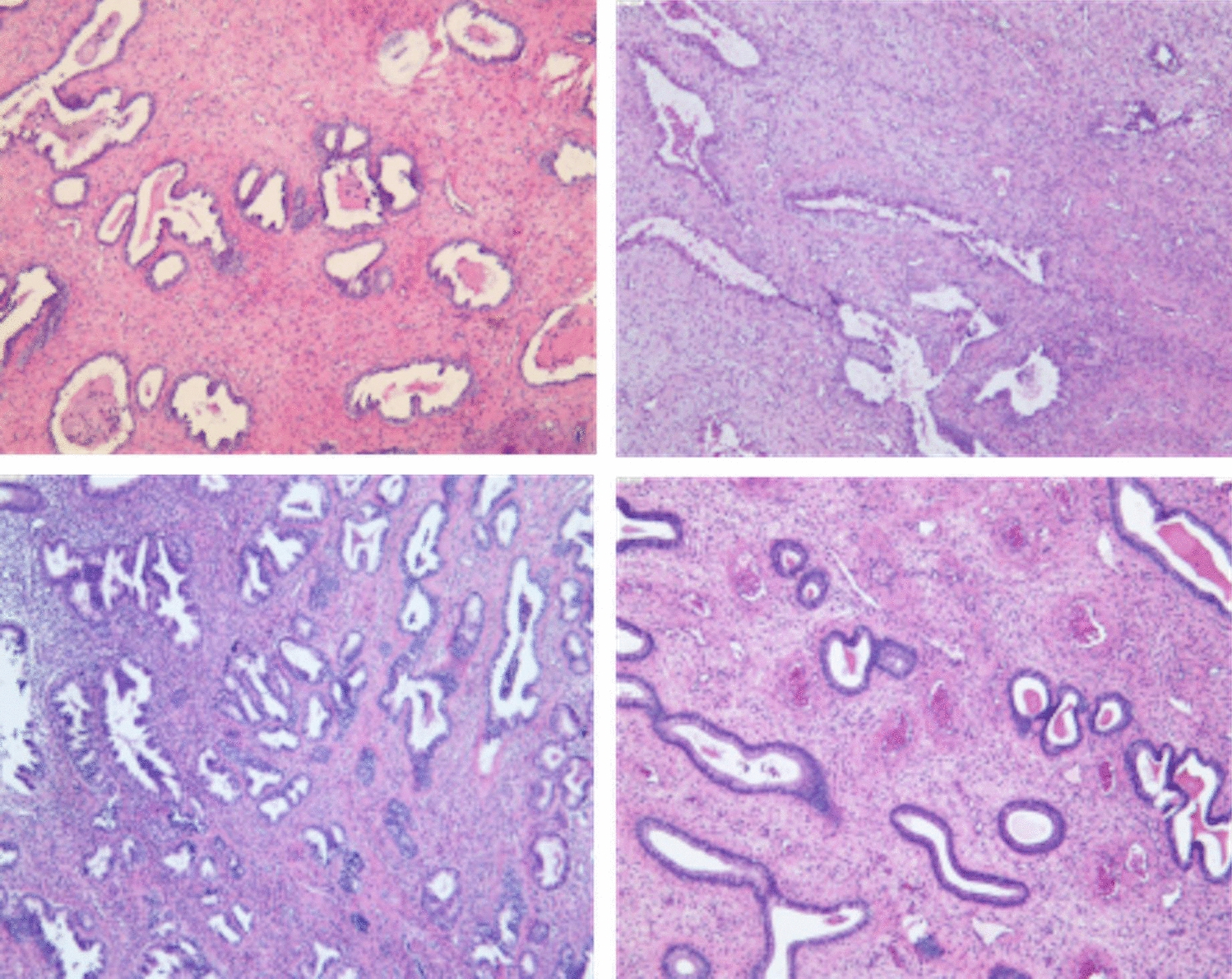
Histological analysis of TPA and EP ((H&E staining, original magnification × 40)

The typical pathological manifestations of endometrial TPA included proliferating and benign endometrial glands. In addition, the stroma around the glands was characterized by spirally staggered bundles of smooth muscle cells (Fig. 3).

### Comparison of recurrence rates between the TPA and EP groups

**Fig. 4:**
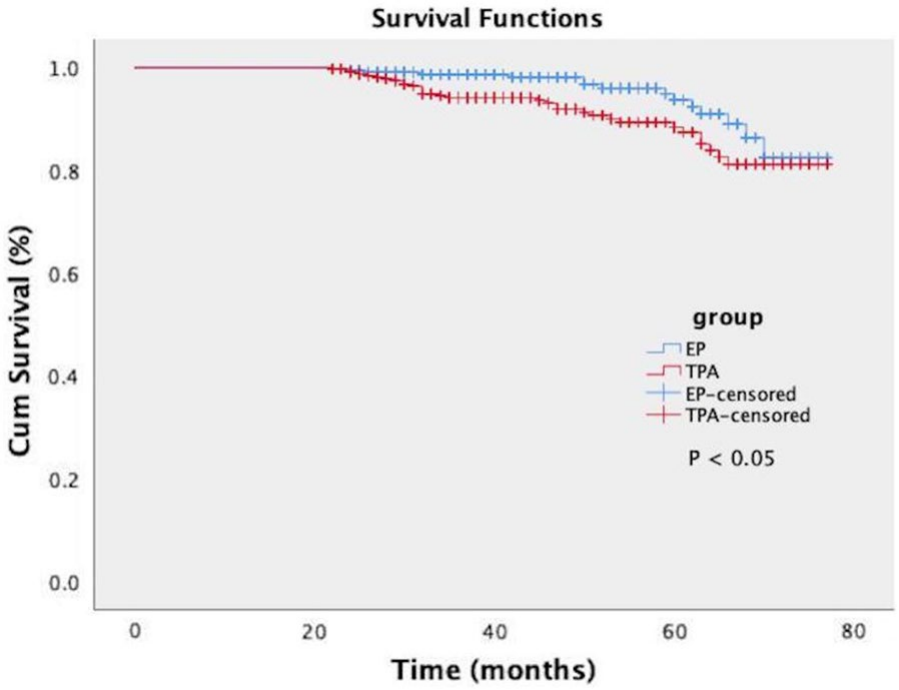
Kaplan–Meier curves for recurrence rate in the TPA and EP groups

360 cases of TPA and 367 cases of EP were followed up for 22–77 months and the median follow-up time was 60.83 months. The recurrence rate in the TPA group was 8.89%; this compared to 3% in the EP group. In the TPA group, 10 cases had abnormal bleeding and 4 cases had menstrual changes. Another 18 cases were asymptomatic. In the EP group, 3 cases had abnormal bleeding and 2 cases had menstrual changes. 1 cases had secondary dysmenorrhea. Another 5 cases were asymptomatic. A log-rank test revealed a significant difference in recurrence rate between the two groups (*P* < 0.05). The recurrence rate in the TPA group was significantly higher than that in the EP group (Fig. 4).

### Risk factors for the recurrence of TPA

Of the 360 TPA patients who underwent conservative surgery, including hysteroscopic electrotomy, curettage, and polyp clamping, 32 cases (8.89%) experienced relapse. Parity, menopausal status, and surgical method were related to the recurrence of TPA. Furthermore, a gravidity > 3, menopause, curettage, and polyp clamping were identified as independent risk factors for the recurrence of TPA (*P* < 0.05; Table 4).

**Table 4:** Univariate and multivariate analyses for risk factors of recurrence (*n* = 32)

Variable	Recurrence rate (%)	Univariate analysis	Multivariate analysis	
		*P*-value	OR (95% CI)	*P*-value
Age (years)				
≤ 50	10.10 (20/198)	0.418	Variable removed	
> 50	7.41 (12/162)			
Menopause				
No	2.83 (3/106)	< 0.001	3.143 (1.986–5.113)	< 0.001
Yes	11.41 (29/254)			
BMI				
Normal	9.43 (10/106)	0.886	Variable removed	
Overweight	8.78 (18/205)			
Obese	8.16 (4/49)			
Gravidity				
≤ 3	3.40 (5/147)	0.020	1.483 (1.345–3.226)	0.029
> 3	12.68 (27/213)			
Parity				
≤ 2	7.64 (12/157)	0.517	Variable removed	
> 2	9.85 (20/203)			
Method of surgery				
Hysteroscopic electrotomy	2.41 (8/332)	0.026	1.429 (1.056–2.968)	0.037
Curettage	80.95 (17/21)			
Polyp clamp	100 (7/7)			

BMI, body mass index; OR, odds ratio; CI, confidence interval

## Discussion

TPA is a rare lesion that occupies the uterine cavity or the cervical canal and consists of fibroid stroma and glands [10]. There was no apparent specificity in terms of clinical manifestations, transvaginal ultrasound, and the tumor morphology of TPA. Thus, this condition is easily confused with other uterine lesions, especially EP. Pathological examination remains the gold standard for a definite diagnosis.

In this study, abnormal bleeding was the most common manifestation and affected 63.11% of patients; this finding was consistent with previous research [11]. Compared to patients in the EP group, patients with TPA were more likely to present with clinical manifestations such as irregular vaginal bleeding, menorrhagia, menostaxis, and dysmenorrhea (*P* < 0.05). The clinical symptoms of TPA were closely related to morphology, anatomical location, and pathological features [12, 13].

On ultrasound images, the disease manifested as a well-defined uterine cavity or a strong or partial echo in the cervical canal in a manner that was similar to other uterine space-occupying lesions [14]; however, these observations lacked specificity. Hysteroscopy revealed that the proportion of TPA patients with a lesion size > 3 cm and multiple polyps was significantly higher than that in the EP group (*P* < 0.05). Moreover, both ultrasound and hysteroscopy showed that when compared with EP, TPA was more likely to exist in the lower segment of the uterine cavity. Large polyps and multiple polyps located in the lower segment of the uterine cavity were more likely to cause abnormal uterine bleeding. The typical pathological manifestations of endometrial TPA were proliferating and benign endometrial glands, and the stroma around the glands was characterized by spirally staggered bundles of smooth muscle cells. These bundles of smooth muscle cells allows polyps to implant in the uterine muscle wall to achieve a better blood supply, thus making it easier to grow into a larger polyp [15].

The pathogenesis of TPA remains unclear. However, some researchers [15, 16] hypothesized that adenomyomatous polyps originate from stromal progenitor cells in the endometrium which can differentiate into smooth muscle and may represent the product of long-term estrogen stimulation. Lin et al. reported a case in which a gonadotropin-releasing hormone agonist effectively reduced the size of the lesion, thus suggesting that growth was dependent on the level of estrogen [17]. In this study, the mean age of TPA group was 53.54 ± 3.19 years. The incidence rate was significantly higher in women > 50 years of age than in women < 50 years of age; this may be related to long-term or continuous estrogen stimulation with little or no progesterone antagonism in this age group. Moreover, tamoxifen has been reported to exhibit a weak estrogen-like effect when administered to postmenopausal women that increases the occurrence and recurrence rate of TPA [18, 19]. In contrast, in the present study, there was no significant difference in the recurrence rate when compared between different age groups after conservative surgery for TPA; thus, age was not a risk factor for the recurrence of TPA. This result may be related to the short follow-up duration and the small sample size of the present study.

However, analysis also showed that the proportion of postmenopausal women in the TPA group was significantly higher than that in the EP group (*P* < 0.05), thus suggesting that postmenopausal women are more likely to develop TPA. In addition, we found that the recurrence rate in postmenopausal women after treatment was higher than that of premenopausal women (*P* < 0.05). Reduced estrogen levels in postmenopausal women can lead to increased levels of follicle-stimulating hormone, thus leading to the increased expression of estrogen receptors in utero [6]. In a previous study, Longacre et al. also reported that in addition to the high estrogen level in patients, the abnormal expression of estrogen receptors in the local endometrium was also associated with TPA occurrence [20]. In the present study, postmenopausal women had a significantly higher BMI than premenopausal women. Overweight and obesity rates were also considerably more significant in postmenopausal women. The BMI of postmenopausal women has been reported to be positively correlated with the levels of estrogen over an extended period [21]. Weight gain and obesity result in insulin resistance and hypertension; research has shown that insulin resistance is closely linked to abnormally high estrogen levels [21]. Therefore, obese individuals are more likely to develop TPA. In the present study, 255 patients (52.25%) were overweight and 87 (17.83%) were obese, thus supporting previous reports relating to the occurrence of TPA.

In the present study, we found that the pathology of TPA was different from that of EP and was characterized by abundant smooth muscle interstitium and endometrial glands, which was similar to that of adenomyosis. Furthermore, it has been reported that TPA occurs when adenomyoma intrudes into the uterine cavity, thus placing TPA under the category of adenomyosis of the uterus [22, 23]. In this study, we found that most patients had a history of pregnancy. Patients with a gravidity > 3 and a parity > 2 were more likely to develop TPA. Furthermore, compared with EP, TPA was more likely to exist in the posterior wall, followed by the anterior wall. The posterior wall and anterior wall are known to be the anatomical locations with the highest rates of embryo implantation [24]. Collectively, these results suggest that surgery of the uterine cavity and endometrial injury may be related to the occurrence of TPA.

In total, 360 patients in the TPA group and 367 patients in the EP group underwent conservative surgery, including hysteroscopic electrotomy, curettage, and polyp clamping. The recurrence rate in the TPA group was 6.56%; this compared to 3% in the EP group. A log-rank test revealed a significant difference in the recurrence rate between the two groups; recurrence rate in the TPA group was significantly higher than that in the EP group (*P* < 0.05) while parity, menopausal status, and surgical method, were all related to the recurrence of TPA. Furthermore, a gravidity > 3, menopause, curettage, and polyp clamping were all identified as independent risk factors for TPA recurrence. These results supported the link between elevated estrogen levels, its receptors and endometrial damage. When planning surgery, it is essential to consider the unique pathogenesis and pathological characteristics of TPA. This type of lesion cannot be directly removed by curettage and clamp, and the postoperative residual rate of lesion is high [22]. Hysteroscopy can remove directly visible lesions, while other suspected endometrial lesions can be biopsied simultaneously with minimal trauma [25]. In the present study, 332 patients who underwent hysteroscopic electrotomy were followed up successfully; only eight patients experienced recurrence without any malignant change, thus indicating that hysteroscopic electrotomy is a safe and effective treatment method. Furthermore, hysteroscopic resection of directly visible lesions could reduce trauma and preserve fertility, thus yielding a definite curative effect [26, 27].

Although the clinical manifestations, ultrasonography and hysteroscopy of TPA lack specificity, for women with risk factors, the possibility of TPA should be fully recognized before surgery, and the depth of incision should be increased during surgery to reduce the recurrence rate. Since the recurrence rate of TPA is higher than that of EP, it is necessary to strengthen postoperative monitoring of TPA patients for early detection of recurrence and timely treatment.

## Conclusions

TPA is a particular type of lesion that occupies the uterine cavity. The typical pathological manifestations of endometrial TPA are proliferating and benign endometrial glands. Furthermore, the stroma around the glands is characterized by spirally staggered bundles of smooth muscle cells. We found that abnormal bleeding was the common manifestation of endometrial TPA. Pathological examination remains the gold standard for definite diagnosis.

Although the pathogenesis of this disease is not fully understood, elevated levels of estrogen levels and its receptors, along with endometrial damage, have all been established as risk factors for the occurrence and recurrence of TPA. However, the specific relationship between these risk factors and disease occurrence still needs to be investigated further. Hysteroscopic electrotomy is the preferred treatment option for endometrial TPA, especially for women with risk factors. The unique pathogenesis and pathology of TPA suggest that increasing the depth of ablation may prevent the recurrence of TPA.

### Limitations

There are some limitations to this study that need to be considered. First, we could not assess all variables that are potentially associated with the occurrence and recurrence of TPA in this single study. Furthermore, the inclusion of cases involving only one hospital might have reduced the external validity of the results. Further prospective studies with a larger sample size in a broader context are needed.

## Data Availability

Not applicable.

## Abbreviations

APA: Atypical polypoid adenomyomas
BMI: Body mass index
EP: Endometrial polyps
TPA: Typical polypoid adenomyoma
